# Metastatic involvement of the spleen in differentiated carcinoma of thyroid

**DOI:** 10.4103/0972-3919.78256

**Published:** 2010

**Authors:** Purushottam Kand, Ramesh Asopa

**Affiliations:** Radiation Medicine Centre, Bhabha Atomic Research Centre, Tata Memorial Hospital Annexe Building, Mumbai, India

**Keywords:** Differentiated thyroid carcinoma, fine needle aspiration cytology, splenic metastasis, 131-I whole body scan

## Abstract

Splenic metastasis in differentiated thyroid carcinoma is rare occurrence. We describe an unusual case of diffuse metastatic splenic involvement with normal hematological indices in differentiated thyroid carcinoma demonstrated by post-therapy whole body radioiodine scan.

Splenic metastasis of Differentiated Thyroid Carcinoma (DTC) is rare. Literature reveals two cases reported by Pauloni *et al*,[1] and Mayayo *et al*.[2] Mohan *et al*, have reported multiple littoral cell angiomas mimicking metastatic thyroid carcinoma to the spleen.[3]

A 50 year old female patient diagnosed follicular variant of papillary carcinoma of thyroid [Figure 1 H and E section - 400×) with skeletal metastasis demonstrated avid 131-I concentration in the thyroid bed with focal uptake in multiple skeletal metastatic sites and diffuse uptake in the entire spleen on the whole body post therapy scan in anterior and posterior views [Figure 2] after 3.515 GBq of 131-I ablation dose. USG guided Pap stained FNAC smear from spleen confirmed thyroid carcinoma cells arranged in follicular pattern [Figure 3]. The patient had normal hematological indices even with diffuse splenic involvement seen on the 131-I scan.

**Figure 1:**
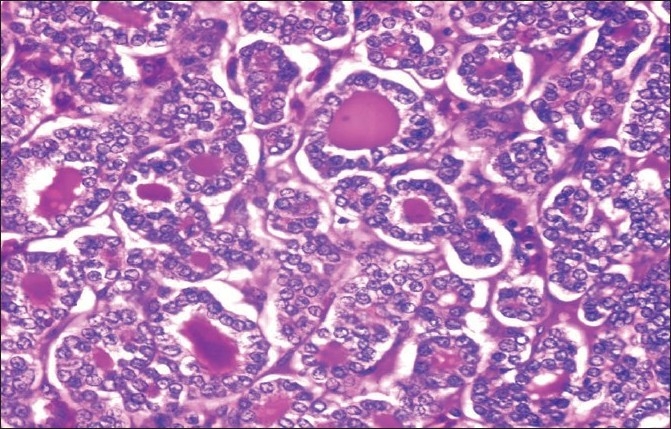
H and E section (400×) of the primary site in thyroid bed demonstrating follicular variant of papillary carcinoma of thyroid

**Figure 2:**
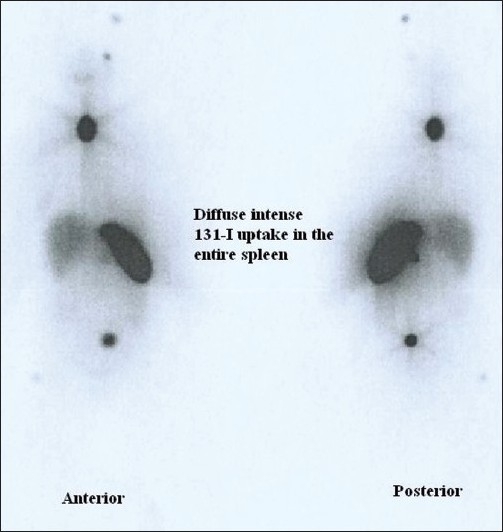
Whole body 131 I post therapy scan demonstrating diffuse intense uptake in the entire spleen with multiple skeletal lesions with focal abnormal tracer concentration

**Figure 3:**
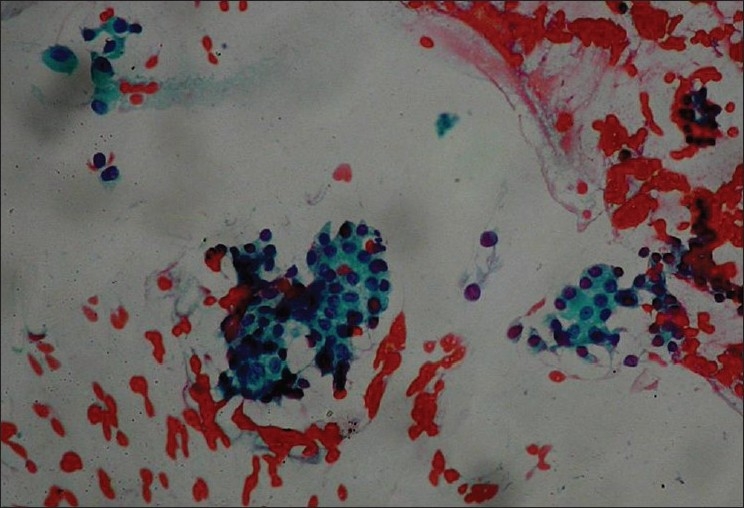
USG guided Pap stained FNAC smear from spleen demonstrating thyroid carcinoma cells arranged in follicular pattern

A year later, no new metastatic foci were noted in the post therapy whole body scan after administration of 8.437 GBq 131-I for the metastatic disease indicating stable disease inspite of splenic involvement. 131-I whole body post therapy scan provides improved detection of local and distant metastatic deposits as compared to low-dose diagnostic studies.[4,5]

Metastatic splenic involvement in DTC is diagnosed with whole body 131-I scan and UGS guided FNAC. Although this marks aggressive spread of the disease, its impact on patient morbidity and mortality remains unexplored due to its rare occurrence.
